# Modulatory effects of cAMP and PKC activation on gap junctional intercellular communication among thymic epithelial cells

**DOI:** 10.1186/1471-2121-11-3

**Published:** 2010-01-15

**Authors:** Oscar K Nihei, Paula C Fonseca, Nara M Rubim, Andre G Bonavita, Jurandy SPO Lyra, Sandra Neves-dos-Santos, Antonio C Campos de Carvalho, David C Spray, Wilson Savino, Luiz A Alves

**Affiliations:** 1 Laboratory of Cellular Communication, Oswaldo Cruz Institute, The Oswaldo Cruz Foundation, Rio de Janeiro, Brazil; 2 Laboratory of Thymus Research, Oswaldo Cruz Institute, The Oswaldo Cruz Foundation, Rio de Janeiro, Brazil; 3 Department of Pathology, Federal University of Rio de Janeiro State (UNIRIO), Rio de Janeiro, Brazil; 4 Department of Clinical Analysis, Faculty of Pharmacy, Federal University of Juiz de Fora, Juiz de Fora, Brazil; 5 Institute of Biophysics Carlos Chagas Filho, Federal University of Rio de Janeiro, Rio de Janeiro, Brazil; 6 The Dominick P. Purpura Department of Neuroscience, Albert Einstein College of Medicine, New York, USA

## Abstract

**Background:**

We investigated the effects of the signaling molecules, cyclic AMP (cAMP) and protein-kinase C (PKC), on gap junctional intercellular communication (GJIC) between thymic epithelial cells (TEC).

**Results:**

Treatment with 8-Br-cAMP, a cAMP analog; or forskolin, which stimulates cAMP production, resulted in an increase in dye transfer between adjacent TEC, inducing a three-fold enhancement in the mean fluorescence of coupled cells, ascertained by flow cytometry after calcein transfer. These treatments also increased Cx43 mRNA expression, and stimulated Cx43 protein accumulation in regions of intercellular contacts. VIP, adenosine, and epinephrine which may also signal through cyclic nucleotides were tested. The first two molecules did not mimic the effects of 8-Br-cAMP, however epinephrine was able to increase GJIC suggesting that this molecule functions as an endogenous inter-TEC GJIC modulators. Stimulation of PKC by phorbol-myristate-acetate inhibited inter-TEC GJIC. Importantly, both the enhancing and the decreasing effects, respectively induced by cAMP and PKC, were observed in both mouse and human TEC preparations. Lastly, experiments using mouse thymocyte/TEC heterocellular co-cultures suggested that the presence of thymocytes does not affect the degree of inter-TEC GJIC.

**Conclusions:**

Overall, our data indicate that cAMP and PKC intracellular pathways are involved in the homeostatic control of the gap junction-mediated communication in the thymic epithelium, exerting respectively a positive and negative role upon cell coupling. This control is phylogenetically conserved in the thymus, since it was seen in both mouse and human TEC preparations. Lastly, our work provides new clues for a better understanding of how the thymic epithelial network can work as a physiological syncytium.

## Background

Intercellular communication mediated by gap junctions has been considered ubiquitous during the development, maturation, homeostasis and death of diverse cell types and tissues in metazoa [1-7]. These junctions are membrane specializations located in cell-cell contact regions, where intercellular hydrophilic conduits, assembled as dodecameric protein complexes, directly connect the cytosols of adjacent cells [8]. Each complex is composed by two hexameric hemichannels, the connexons, one in each cell [9,10]. In vertebrates, members of the connexin protein family form these channels, which in rodents has at least 20 isoforms [11,12]. Topologically, the connexin protein contains four hydrophobic transmembrane domains (M1 to M4), two conserved extracellular loops (E1-E2), one intracellular loop and intracellular C- and N-terminal domains [13]. With an estimated permeability limited to molecules below approximately 1 kDa, these intercellular channels allow cells to share metabolites such as glucose and nucleotides, buffer ions such as K^+ ^and H^+^, and convey important intracellular second messengers such as calcium, cyclic 5'-adenosine monophosphate (cAMP) and 1,4,5-inositol-trisphosphate (IP3) [14-18]. Physiologically, gap junctions have been associated with diverse phenomena such as transmission of electrical signals (as electrotonic synapses) and intercellular calcium waves, metabolic and ionic coupling, and cellular synchronization [19-22]. In this respect, loss or dysfunction of gap junctions have been related to distinct diseases [23-28].

Gap junction channels may be modulated at different levels. Gap junction channel gating, i.e., shifting between open and closed states, is regulated by voltage, intracellular pH (pHi) and Ca^2+ ^([Ca^2+^]i), and phosphorylation [29-31]. It has been suggested that the connexin C-terminal and the intracellular loop of the protein are associated with gap junction channel sensitivity to pHi and [Ca^2+^]i, while the M1 domain, the N-terminal and the E1 domain have been associated with the voltage sensor [13,29]. The C-terminal domain exhibits diverse kinase recognition motifs, which allow channel regulation by threonine/serine and tyrosine kinases.

Functional GJIC has been shown in a variety of cell types of the immune system, such as T and B lymphocytes, dendritic cells, microglia, monocytes, macrophages, neutrophils and mast cells [32-38]. In vitro experiments have demonstrated Cx43 mediated functional GJIC between thymic epithelial cells [39,40]. In addition, data obtained from Cx43^-/- ^mice revealed that this protein is important to normal T cell lymphopoiesis [41].

Despite the multiple possibilities of regulation of thymic physiology by diverse neuroendocrine products [42], few previous studies have evaluated GJIC modulation in thymic epithelial cells. Head et al. [43,44] demonstrated, by dye injection, that treatment of thymic epithelial cells with soluble factors such as interleukin-1 (IL-1), growth hormone (GH), adrenocorticotrophic hormone (ACTH), steroid hormones and neuropeptides induced a partial inhibition of coupling and in some cases it diminished thymulin secretion.

The modulation of GJIC may also be evaluated through the activation of different intracellular signaling pathways by specific second messenger analogs, as well as agonists or antagonists of relevant signaling molecules. The importance of cAMP and PKC in mediating intracellular signaling of diverse extracellular messengers is widely recognized [45,46]. cAMP activates cAMP-dependent protein kinase (PKA) [47]; and PKC is activated by diacylglycerol and/or calcium (or neither depending on its isoform), as a result of phospholipid signaling pathways [48]. The effects of cAMP on GJIC have been investigated in systems such as hepatocyte primary cultures, cardiac myocytes, ovarian follicles, myometrium, carotid body and retina [15,49-53]; and in cell lines derived from ovarian granulosa cells, endometrial and colonic epithelium, endothelium, osteoblasts, and mammary tumor cells [54-57].

The effects of PKC activation on GJIC have also been investigated [31]. In general, cAMP acts by enhancing GJIC while PKC inhibits it [49,51,57-59]. However, contrasting results have been reported for both signaling pathways [15,50,53,56,60]. Nevertheless, to our knowledge the effects of cAMP elevation or PKC activation on GJIC have not been studied in the immune system. Herein we investigated the putative modulatory effects of cAMP and PKC on GJIC between thymic epithelial cells (TEC). Our results demonstrate that inter-TEC GJIC is upregulated by cAMP and downregulated by PKC.

## Results

### Evaluation of functional GJIC-mediated dye transfer by flow cytometry

**Figure 1:**
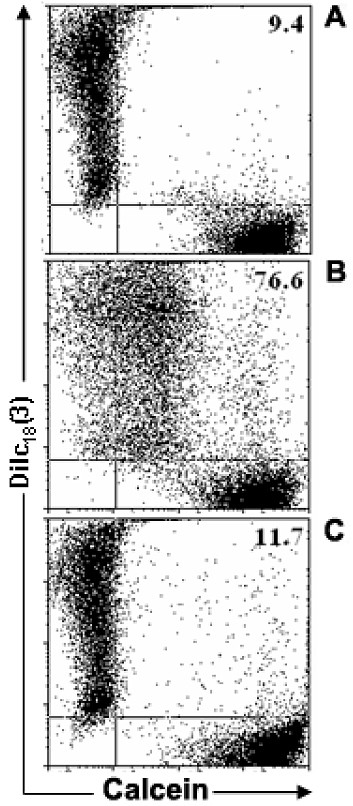
**Flow cytometric analysis of the mouse thymic epithelial cell line, showing inter-TEC gap junction intercellular communication**. Calcein^+^Dilc_18_(3)^- ^and calcein^-^DiIc_18_(3)^+ ^IT-76M1 cells were co-cultured for 6 hr at 37°C. These cells were then dissociated and analyzed by flow cytometry to quantify the double positive [calcein^+^Dilc_18_(3)^+^] cells. Some calcein^+^Dilc_18_(3)^- ^and calcein^-^DiIc_18_(3)^+ ^cells were separately cultured and used to adjust the cytometry settings. These cells also were used to establish the control population (**A**). Data are presented in the form of dot plots (**A**, **B**, **C**), which depict two-dimensionally the labeling pattern of each cell population considering the fluorescence intensity (log scale) of calcein and DiIc_18_(3). In **B**, the 6 hr co-cultured cells are shown, where the presence of double positive cells is apparent, indicating the dye coupling. In **C**, cells co-cultured for 6 hours in the presence of 18-β-glycyrrhetinic acid (GRA; 100 μM) exhibited a complete inhibition of inter-TEC GJIC. These data are representative of at least 4 experiments.

Confirming previous results from our Laboratory [39,61,62] flow cytometry experiments revealed that after 6 hours of co-culture more than 65% of the initial single positive DiIc_18_(3)^+ ^TEC acquired calcein (Figure 1B). To ascertain that gap junctions mediated this dye transfer we treated the cells for 6 hrs with 100 μM of 18-β-glycyrrhetinic acid (GRA), a gap junction inhibitor. Such procedure inhibited inter-TEC GJIC by >85% (Figure 1C). Similar results were also obtained when the epithelial were treated with carbenoxolone, another gap junction inhibitor (data not shown).

### cAMP elevation enhances inter-TEC GJIC

**Figure 2:**
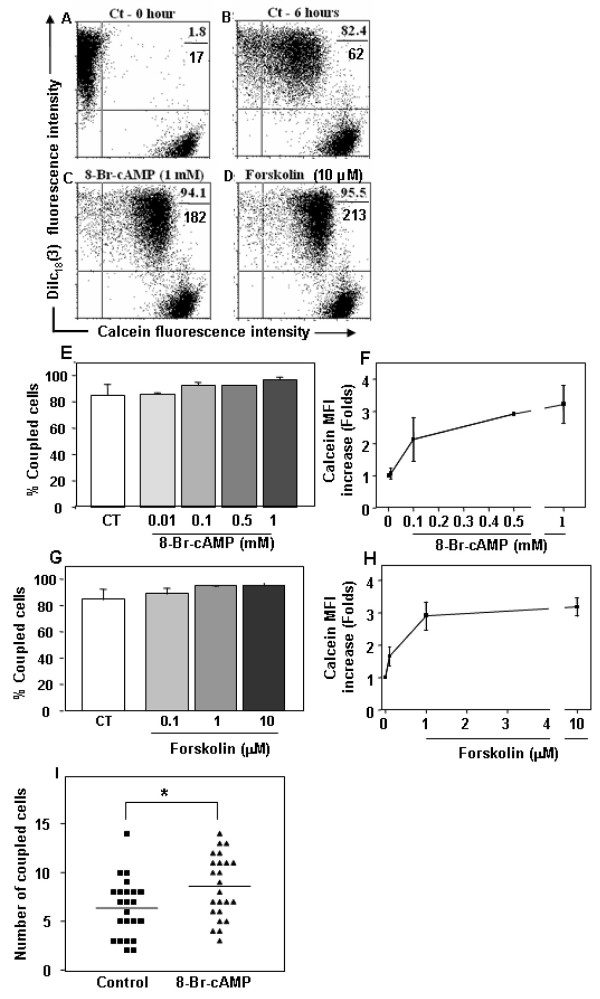
**Increase in cAMP enhances inter-TEC GJIC**. Panels **A **and **B **depict untreated co-cultures of the mouse TEC line at zero and 6 hours time points, respectively. The percentage of double positive cells and the calcein geometric mean fluorescence intensity (MFI) of these populations are depicted at the upper right corner of each panel (%, above; MFI, below). Panels **C **and **D **show the inter-TEC coupling, following 6 hours of treatment with 1 mM 8-Br-cAMP or 10 μM forskolin. Both treatments enhanced the calcein mean fluorescence intensity of coupled cells (double positive cells). These data are representative of at least 4 separate experiments. Such enhancements can also be seen in Panels **E **to **H**, depicting TEC co-cultures treated for 6 hours with increasing concentrations of either 8-Br-cAMP (**E**-**F**) or forskolin (**G**-**H**). While percentages of coupled TEC was not significantly modified (**E, G**), the geometric mean fluorescence of calcein quantified from the double positive cells tripled after both treatments (**F, H**). The results are representative of 3 independent experiments (mean SD). Panel **I **shows that 8-Br-cAMP was also capable of enhancing inter-TEC GJIC, in primary cultures of human TNC-derived epithelial cells. Numbers of coupled cells were count in blind. * p < 0.05.

To analyze the putative modulatory effects of cAMP on GJIC in TEC cultures, we stimulated the mouse TEC line with 8-Br-cAMP, a membrane permeable cAMP analog; or forskolin, which stimulates cAMP production through activation of adenylate cyclase isoforms [63]. For that, TEC co-cultures were either treated or not with 8-Br-cAMP (1 mM) or forskolin (10 μM) for 6 hrs and subsequently analyzed by flow cytometry. Two distinct quantitative parameters were calculated comparing the treated versus control double positive TEC: the extent of cell coupling (% double positive cells) and the cell coupling efficiency (based on calcein geometric mean fluorescence). Under these conditions, the extent of coupling was not significantly modified, ranging from 85.0 ± 8.2% (Mean ± SD) at control level to up to 96.7 ± 1.9% and 95.5 ± 2.2% with 8-Br-cAMP and forskolin, respectively, indicating that the TEC monolayer was functionally well coupled under control confluent culture conditions (Figure 2A-D). Nevertheless, treatment with 8-Br-cAMP or forskolin increased transfer efficiency, inducing respectively, 3.21 ± 0.58 and 3.18 ± 0.29 fold increase in geometric mean fluorescence of calcein labeling in double positive cells (Figure 2A-D). The half maximal effective concentration (EC_50_) for 8-Br-cAMP and forskolin was 98 μM and 0.470 μM, respectively (Figure 2E-H). Importantly, upregulation of inter-TEC GJIC triggered by 8-Br-cAMP was not restricted to the mouse TEC line, since similar results were seen when TNC-derived primary cultures of human TEC (Figure 2I) and IT76 M1 cells (data not shown) were treated with 8-Br-cAMP, as ascertained after lucifer yellow microinjection and blind evaluation of the numbers of coupled cells.

We observed the same effect when the intracellular dye microinjection assay was performed on IT76M1 cells.

**Figure 3:**
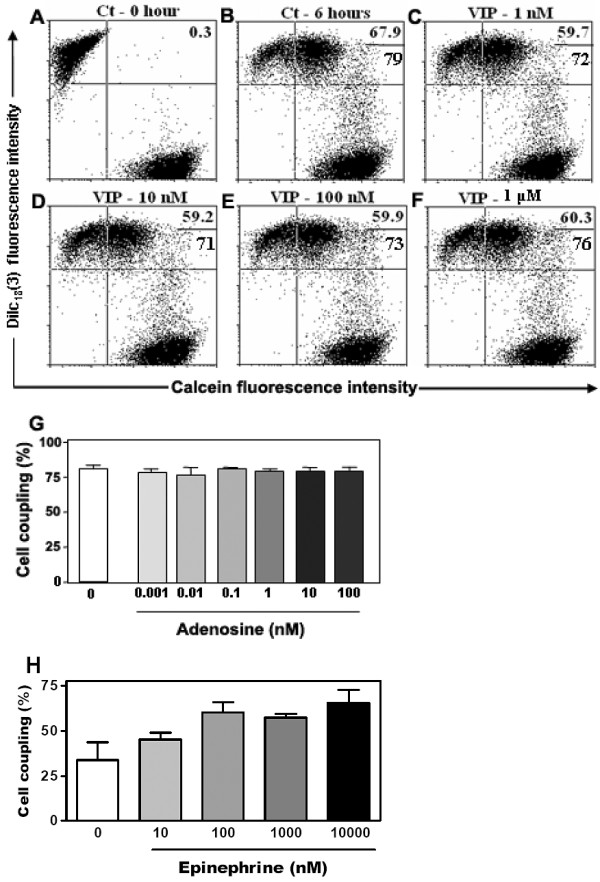
**Vasoactive intestinal peptide (VIP), adenosine and epinephrine effects on basal levels of inter-TEC GJIC**. Co-cultures of the mouse TEC line were either treated or not (Ct- 6 hrs) with increasing concentrations of VIP (1 - 1000 nM, panels **C **to **F**), and the degree of cell coupling, ascertained by cytofluorometry, did not change as compared to the 6 hours untreated control (**B**), in relation to both percentages of coupled cells and calcein mean fluorescence. Values are shown at the upper right corner of each panel, representing the percentage of double positive cells (above) and the calcein geometric mean fluorescence intensity (below) of these populations. Panel **A **depicts the flow cytometry profiles of TEC that were not co-cultured. Panel **G **shows that adenosine does not alter inter-TEC GJIC as well, as revealed by the percentages of coupled cells seen after treatment of various doses of the nucleotide. Panel **H **shows that epinephrine (1 nM to 10 μM) increased the percentage of dye coupling between TEC in a dose-dependent fashion. Data are shown as mean ± standard deviation, being representative of 2 independent experiments performed in triplicate.

We also tested if inter-TEC GJIC could be modulated by distinct extracellular messenger molecules, which also signal through cyclic nucleotides. For that we applied the vasoactive intestinal peptide (VIP), adenosine and epinephrine. VIP^+ ^nerve terminals and VIP receptors have been characterized in thymic parenchyma [64,65], whereas adenosine, which activates P1 receptors, has been implicated in thymocyte death [66,67] and epinephrine, a systemic catecholamine, whose actions include control of TEC cytokine secretion [68]. We treated mouse TEC line cultures with increasing concentrations of VIP (1 nM to 1 μM), adenosine (1 nM to 100 μM) and epinephrine (100 nM to 1 μM). Neither VIP (Figure 3A-F) nor adenosine (Figure 3G) changed in the extent of inter-TEC GJIC. However, treatment with the adrenoreceptor agonist epinephrine induced an increase on dye coupling in a dose-dependent manner (Figure 3H).

### cAMP elevation upregulates Cx43 at different levels

**Figure 4:**
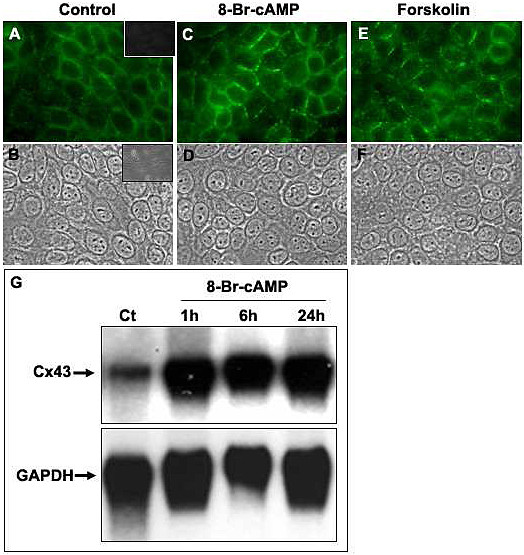
**Increase in cAMP enhances connexin gene and protein expression**. Cultures of the mouse TEC line were treated with either 8-Br-cAMP (1 mM) or forskolin (10 μM) for 6 hrs at 37°C. Cells were then fixed, permeabilized and labeled with the anti-Cx43 polyclonal antibody, ultimately revealed with the Alexa 488-conjugated secondary antibody. The fluorescence microscopy images (**A**, **C**, **E**) and the corresponding phase contrast images are depicted (**B**, **D**, **F**). The mouse TEC treated with 8-Br-cAMP (**C**, **D**) and forskolin (**E**, **F**) presented an increased punctate labeling of Cx43, mainly at cell-to-cell contact regions, when compared with the untreated controls (**A**, **B**). Inserts in **A **and **B **show the fluorescence microscopy image and respective phase contrast image of TEC subjected to isotype control primary antibody and Alexa-488-coupled secondary antibody (Magnification, ×400). Panel **G **show by northern blot analysis that 8-Br-cAMP also enhances Cx43 gene transcription. Mouse TEC were treated or not (Ct) with 8-Br-cAMP (1 mM) and cultured for 1, 6 or 24 hrs at 37°C. The total RNA was then extracted, and 10 - 20 μg of total RNA was loaded in 1.2% agarose gel and plotted onto nylon membrane. For detection of Cx43 mRNA, the membrane was hybridized with a ^32^P-labeled cDNA probe for Cx43. Alternatively, the membrane was also hybridized with a ^32^P-labeled cDNA for GAPDH. The amount of Cx43 mRNA in IT-76M1 cells was increased after 1, 6, and 24 hours of treatment with 8-Br-cAMP.

We also investigated the possible mechanisms underlying the GJIC stimulatory effects of cAMP elevation. When mouse TECs were evaluated by immunofluorescence, the labeling pattern generated with the anti-Cx43 antibody revealed that both 8-Br-cAMP and forskolin induced an accumulation of Cx43 protein at regions of intercellular contacts, presenting a punctate pattern of distribution (Figure 4A-F). The northern blot analysis revealed an increase in Cx43 mRNA as early as 1 hr after the treatment with 8-Br-cAMP, which continued to be observed after 6 and 24 hours of treatment (Figure 4G).

### Phorbol ester induced PKC activation and down-regulates inter-TEC GJIC

**Figure 5:**
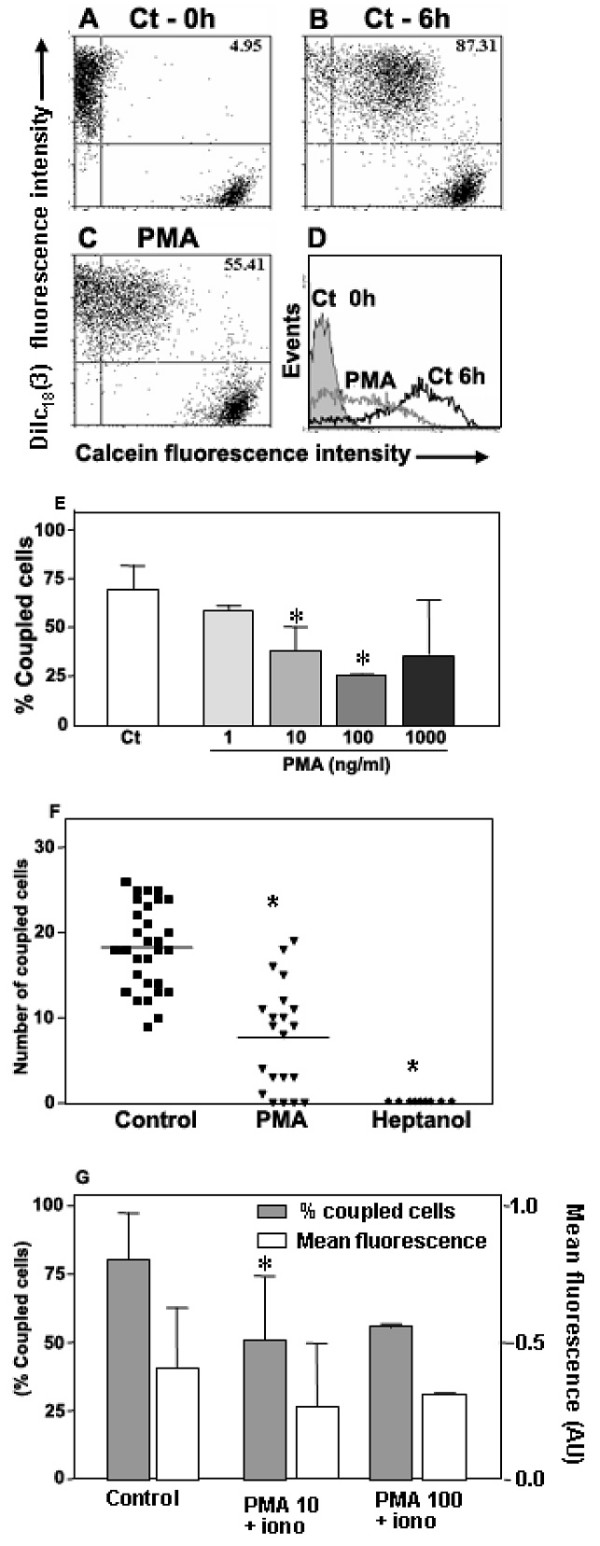
**Phorbol myristate acetate inhibits GJIC in mouse and human TEC**. Panels **A **to **D **are flow cytometry profiles showing mouse TEC co-cultures that were either treated (**C**) or not (**B**) with PMA (10 ng/ml) and maintained for 6 hrs at 37°C. Panel **A **represents the TEC population, which was separately cultured (Ct - 0 hours). The percentage of double positive cells is depicted at the upper right corner of each panel. The histograms with the calcein fluorescence profile of each population are depicted in panel **D**: calcein^-^Dilc_18_(3)^+ ^cells, not submitted to co-culture (gray filled profile); control co-cultured TEC (black line); co-cultured TEC treated with PMA (gray line). Data are representative of at least 4 experiments. Panel **E **shows a dose-response curve of the effect of PMA treatment upon inter-TEC GJIC. A significant dose-dependent inhibition of cell coupling is seen, with a plateau being reached in 100 ng/ml. (* p < 0.05). Panel **F **shows that PMA also down-regulates GJIC in primary cultures of TNC-derived human TEC. Panel **G **shows that simultaneous treatment with PMA and ionomycin also significantly inhibited dye coupling among TEC (* p < 0.05). Co-cultures of the mouse TEC line were treated simultaneously with PMA (10 or 100 ng/ml) and ionomycin (1 μg/ml) for 6 hrs at 37°C, analyzed by flow cytometry. The percentage of coupled cells and the calcein geometric mean fluorescence obtained from double positive cells (Mean ± SD) are shown. The data are representative of two independent experiments performed in triplicate.

PKC is a serine/threonine protein kinase that phosphorylates gap junctions and these events have been correlated with the reduction of gap junction communication [69]. Consistent with this finding, and in contrast to cAMP, when mouse TECs were treated with PMA (which activates PKC), a decrease of dye coupling was observed, reducing from 69.26 ± 12.29% at control conditions to 37.74 ± 12.42% and 25.77 ± 0.014% with PMA at 10 and 100 ng/ml, respectively (Figure 5A-F), which represents a dye coupling inhibition of up to 60%. The remaining coupled TEC was not significantly affected after PMA treatment (data not show). A similar down regulation of inter-TEC GJIC was seen in TNC-derived human TEC primary cultures (Figure 5F). Herein we applied microinjection of Lucifer yellow, followed by blind counting of coupled cells. Confirming the participation of gap junctions in this process, 5 mM heptanol completely inhibited dye coupling among human TEC (Figure 5F).

Since the phospholipid signaling frequently induces both PKC activation and [Ca^2+^]i elevation, we also treated the mouse TEC line with both PMA and the calcium ionophore ionomycin (1 μg/ml). In these conditions, a partial but statistically significant inhibition of dye coupling was still seen (Figure 5G).

### Inter-TEC GJIC is not modulated by the presence of thymocytes

Since thymocytes play an important role in TEC differentiation and organization during ontogeny of the thymus [70,71], we investigated if the contact with thymocytes could modulate GJIC in the thymic epithelium.

**Figure 6:**
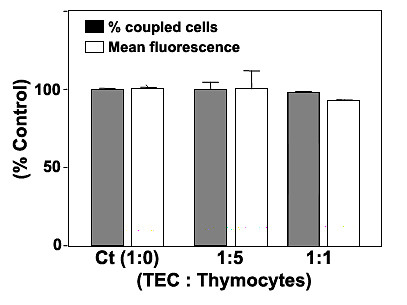
**The presence of thymocytes do not change inter-TEC GJIC**. Mouse TEC co-cultures (IT-76M1 cells) were maintained at 37°C for 2 hrs to establish an adherent confluent monolayer, and then simultaneously cultured with murine thymocytes at 1:5 or 1:10 (TEC:thymocytes) proportion for additional 5 hrs. After this incubation the thymocytes were discarded and epithelial cells were dissociated and analyzed by flow cytometry. The normalized dye coupling degree (gray columns) and the calcein mean fluorescence (white columns) of the double positive cells, clearly show the presence of thymocytes did not significantly modify the levels of dye coupling and dye transfer efficiency among mouse TEC. Data are expressed as mean SD, being derived from obtained from three independent experiments.

After the adhesion and establishment of mouse TEC co-cultures [containing calcein^+^DiIc_18_(3)^- ^and calcein^-^DiIc_18_(3)^+ ^cells], the thymocytes were added at 5 fold or 10 fold excess over TECs. After an additional 5 hours, the thymocytes were discarded and the TEC co-cultures were dissociated and analyzed by flow cytometry. In these conditions, the dye coupling between adjacent TEC was not significantly modulated by thymocytes, neither at 1:5 nor at 1:10 TEC:thymocytes proportions (Figure 6). The calcein fluorescence intensity of calcein^+^DiIc_18_(3)^+ ^was not significantly modified as well (Figure 6).

## Discussion

In the present study we demonstrated, by various experimental approaches, that cAMP and PKC are involved in the modulation of inter-TEC GJIC: the cAMP agonist 8-Br-cAMP enhanced inter-TEC coupling whereas PMA-induced PKC activation triggered an opposite effect.

Using flow cytometry we first detected in a mouse TEC line, that up to 90% of DiIc_18_(3)^+ ^cells co-cultured in 1:1 ratio with calcein+ cells became double positive, a phenomenon which was readily inhibited by the gap junction inhibitors 18-β-glycyrrhetinic acid and carbenoxolone. This result clearly demonstrates that the thymic epithelium spontaneously forms a GJIC-dependent functional syncytium *in vitro*. Under these conditions, 8-Br-cAMP and forskolin did not significantly modify this spontaneous percentage of coupled cells. However, both compounds enhanced up to 3 fold the calcein fluorescence intensity of the double positive cells, indicating an increase in the rate of dye transfer among coupled cells. Importantly, 8-Br-cAMP also induced an increment in GJIC in TNC-derived primary cultures of human TEC. These data clearly show that elevation of cAMP upregulates inter-TEC GJIC, similar to what has been reported for effects on cell types in other systems [51,55].

Experiments performed with the mouse TEC line revealed that 8-Br-cAMP and forskolin treatments induced an accumulation of Cx43 protein at cell-to-cell contact regions, seen as punctate clusters. Furthermore, 8-Br-cAMP induced an increase in Cx43 gene transcription, suggesting that the protein accumulation may be a consequence of altered Cx43 protein translation. Such an alteration in connexin synthesis and accumulation in the cell membrane is in accordance with previous description of modulation of gap junctions by cAMP elevating agonists after long-term evaluations (2-24 hrs, and 7 days) [51,52,54,57,72-74]. However, short-term modulation also has been demonstrated, with description of changes in gap junctional conductance within minutes of treatment with 8-Br-cAMP [75-77]; in some cell types, changes in dye coupling were also detected within 2-10 minutes after this treatment [55,56,77]. In cases of both short- and long-term evaluations alteration in the degree of connexin phosphorylation has also been demonstrated [73,76]. Interestingly, some of these reports have demonstrated that connexin isoforms other than Cx43 also may be regulated by cAMP analogs, such as Cx26, Cx40 and possibly Cx32 [49,56,76,77]. Thus, we cannot exclude the possibility that other connexin isoforms, not yet characterized in TEC, might also be regulated by 8-Br-cAMP and forskolin in IT-76M1 cells.

We also investigated whether physiological stimuli, could signal through cyclic nucleotides, might be involved in modulation of inter-TEC GJIC. When we evaluated VIP and adenosine, we did not observe any change in the degree of cell coupling in the mouse TEC preparation in any concentration of each molecule used. Similar results were seen despite the various concentrations applied for each molecule.

We should mention however, that our finding on VIP is at variance with the data reported by Head and co-workers [44]. These authors reported an inhibition of GJIC in a rat TEC. Although by now we cannot explain such a difference, it may be related to cell line variations or presumably due to receptor signaling dynamics that cannot be merely mimicked by a single agonist [78], or may be related to differences in methodological approaches given that the authors treated the cells before GJIC formation.

In any case, adenosine, which may also signal through cyclic nucleotides depending on its concentration and the activated P1 receptor [67], did not modify the basal level of inter-TEC GJIC as well. These results demonstrate that VIP and adenosine possibly are not physiological modulators of GJIC among TECs. A recent review indicates that adrenoreceptor agonists mediate thymus homeostasis and local T cell development [79]. In addition, reports have suggested a role for catecholamines on thymic epithelial cells controlling proliferation and cytokine secretion [68,80]. Our data demonstrate that epinephrine, and activation of cAMP, lead to an increasing dye coupling in TEC cells suggesting that this mediator could endogenously control communication between thymic epithelial cells and contribute to thymus physiology. We are currently investigating other endogenous molecules which also signal through cAMP, in order to evaluate its potential to modulate inter-TEC GJIC.

In a second set of experiments, we demonstrated that PKC activation (induced herein by the phorbol ester, PMA) significantly inhibits the dye coupling in both mouse and human TEC *in vitro *models.

The fact that the same inhibitory effect was seen in a mouse TEC line as well as in primary cultures of human TEC deserve further discussion. The study by Chanson et al. [56] demonstrated that GJIC inhibition was induced by phorbol esters in a liver-derived cell line but not in differentiated primarily cultures of pancreatic exocrine cells. Similarly, the inhibitory effect of phorbol esters on GJIC was observed in primary cultured developing lens cells, which express Cx43 and Cx49, but not in lentoid cells (differentiated lens cells), which express Cx46 and Cx49, demonstrating that the effects of these compounds might be dependent on the cellular differentiation stage and its pattern of Cx expression [81].

By contrast, the presence of thymocytes apparently is not involved in the control of inter-TEC GJIC, at least in the co-culture experimental conditions that we used. Nevertheless, further studies are still necessary in order to completely discard a role for thymocytes in the control of inter-TEC communication mediated by gap junctions.

## Conclusions

In summary, our data strongly indicate that cAMP and PKC intracellular pathways are involved in the homeostatic control of the gap junction-mediated communication in the thymic epithelium, exerting respectively a positive and negative role upon cell coupling. Importantly, this control is phylogenetically conserved in the thymus, since it was seen in both mouse and human TEC preparations.

In a second vein, we showed that two other extracellular messenger molecules, which also signal through cyclic nucleotides, VIP and adenosine, did not mimic the positive action of 8-Br-cAMP, but epinephrine was able to reproduce this effect, suggesting a specificity control of cAMP in the mechanism of inter-TEC GJIC.

Overall, our work provides new clues for a better understanding of how the thymic epithelial network can work as a physiological syncytium.

## Methods

### Chemicals

Lucifer yellow, triton X-100, 18-β-glycyrrhetinic acid (GRA), forskolin, 8-bromoadenosine 3',5'-cyclic monophosphate (8-Br-cAMP), phorbol 12- myristate 13-acetate (PMA) and ionomycin were purchased from Sigma Chemical Co. (St. Louis, MO, USA). Calcein-AM and DiIc_18_(3) dyes were obtained from Molecular Probes (Eugene, OR, USA), and heptanol was from Merk (Darmstadt, Germany). Collagenase A, dispase II and DNAse grade II were purchased from Boehringer Mannheim Biochemicals (Indianapolis, IN, USA), whereas fetal calf serum was from Hyclone Laboratories (South Logan, UT, USA).

### Thymic epithelial cultures

The mouse TEC line, IT-76M1, was obtained from BALB/c-derived thymic stromal cells after continuous culture. The epithelial nature of this line was ascertained by the presence of desmosomes and cytokeratin filaments [82-84]. These cells were routinely maintained in culture with RPMI 1640 medium supplemented with 10% fetal bovine serum, at 37°C in a 5% CO2 atmosphere.

In addition to the mouse TEC line, we used in some experiments, primary cultures of human TEC, obtained after isolation of thymic nurse cells complexes. For that, fragments of human thymus were obtained from children subjected to cardiac surgery, following the guidelines of the Oswaldo Cruz Foundation's ethics committee. Thymic nurse cells (TNC) are thymic lymphoepithelial complexes that harbor a variable number of thymocytes [85]. When settled in culture, TNCs gradually release thymocytes, and after 3-5 days, a thymocyte-free primary culture of epithelial cells is established. The TNC isolation was performed according to the procedures currently done in our laboratory [86]. In brief, human thymic fragments were minced (~1 mm^3^) and gently agitated for 20 min in RPMI 1640 medium. Released thymocytes were discarded and the thymic fragments were suspended in collagenase A solution (0.2 mg/mL) and further agitated at room temperature (RT) for 20 min. The supernatant was again discarded and the remaining fragments were dissociated enzymatically with a CDD solution (collagenase A - 0.2 mg/mL; dispase II - 0.2 mg/mL; DNAse grade II - 5 μg/ml) for 20 min at 37°C. The digestion product was centrifuged and the pellet suspended in PBS. These cell suspensions were carefully layered above 10 mL of fetal calf serum (Cultilab, Campinas, Brazil) placed in 15 mL tubes. The typically heavier TNCs were allowed to sediment. The TNCs obtained by this process were cultured, and after complete thymocyte release, resulting epithelial cultures were used in all experiments.

### Immunofluorescence

To evaluate connexin expression, TEC were cultured on glass coverslips until confluence. The cells were fixed in cold (-20°C) methanol and further permeabilized by a solution containing 0.2% Triton X-100. The cells were then incubated overnight at 4°C with anti-connexin 43 rabbit polyclonal antibody (Zymed Laboratories, South San Francisco, CA, USA). After washing, the cells were incubated for 1 hour at room temperature with appropriate alexa 488-conjugated secondary antibody (Molecular Probes, Eugene, OR, USA) to reveal the specific labeling. The cells were washed twice, and the coverslips were mounted in PBS-glycerol (3:1) containing 0.1% para-phenylene diamine, an anti-oxidation agent. Cells were analyzed in a Nikon Eclipse TE-300 microscope with phase-contrast and epifluorescence optics, and photographed using a SPOT-RT digital camera (Diagnostic Instruments, Sterling Heights, Michigan, USA).

### Dye microinjection assay

The evaluation of GJIC by intracellular dye microinjection assay was performed as previously described [39]. Human TNC-derived epithelial cells and IT76 M1 cells were cultured in small Petri dishes until confluence. Visualization of cells was performed with an inverted microscope equipped with epifluorescence optics (Axiovert 100, Carl Zeiss, Oberkochen, Germain). The glass microelectrodes were pulled from borosilicate glass (World Precision Instruments, New Haven, CT, USA) using a pipette puller (model PC-10, Narishige, Tokyo, Japan), filled with lucifer yellow (50 mg/ml in 150 mM LiCl) and positioned using a three-dimensional micromanipulator model (MMO-203, Narishige, Tokyo, Japan). Cells were then iontophoretically microinjected with lucifer yellow through brief current pulses. After 1 minute, the number of adjacent cells that acquired the lucifer yellow was quantified. To ascertain that the dye coupling was mediated by GJIC, in some experiments human TEC were also treated with heptanol (5 mM), a gap junction inhibitor.

### Cytofluorometry

GJIC was evaluated using flow cytometry as previously described [61,87]. After confluence, one sample of the mouse TEC line was loaded with calcein-AM (0.5 M) for 30 minutes, while the other was labeled with the lipophilic molecule Dilc_18_(3) (10 μM) for 1 hour. Cells were then washed 5 times with PBS and enzymatically dissociated. Calcein^+^DiIc_18_(3)^- ^cells and calcein^-^DiIc_18_(3)^+ ^cells were co-cultured at 1:1 ratio, being treated (during the co-culture period of 6 hours) or not with drugs of interest. Thereafter, cells were dissociated and the double positive cells [calcein^+ ^DiIc_18_(3)^+^] were quantified by flow cytometry, using a FacsCalibur device (Becton-Dickinson, Mountain View, CA, USA). Double-positive cells thus corresponded the functional calcein transfer from calcein^+^DiIc_18_(3)^- ^to calcein^-^DiIc_18_(3)^+ ^cells. At least 10^4 ^cells were acquired in each experimental condition. In some experiments, TEC were also co-cultured with freshly isolated thymocytes. For this purpose, thymuses were obtained from BALC/c mice, maintained at the Oswaldo Cruz Foundation Animal Facilities (Rio de Janeiro, Brazil).

### RNA extraction and Northern Blotting

Total RNA was extracted from the mouse TEC line, using TRIzol Reagent (Gibco/BRL, Grand Island, NY, USA). For northern blots, 10-20 μg of total RNA was loaded in 1.2% agarose gel containing 0.12 mg/L ethidium bromide. Gels were blotted onto a nylon membrane and fixed by ultraviolet light. For detection of Cx43 mRNA, membranes were pre-hybridized for 1 hr at 65°C in rapid hybridization buffer. Then the membrane was hybridized for 2 hrs at 65°C with rapid hybridization buffer with a ^32^P-labeled cDNA probe for Cx43 or GAPDH. Membranes were washed once in 2× standard saline citrate buffer containing 0.1% SDS at room temperature, then exposed to X-ray film.

### Statistics

For statistical comparisons we applied the two- tail paired Student's *t *test, implemented by the GraphPad Prism software. Differences with p < 0.05 were considered significant.

## Authors' contributions

OKN - designed and performed flow cytometer and dye injection experiments, RNA extraction and Northern blotting experiments, analysed data and drafted the manuscript. PCF - designed and performed experiments. NMR - participated with expertise and participated in writing of the manuscript. AGB - participated with expertise, constructed the figures and participated in writing and review of the manuscript. JSPOL - designed and performed experiments. SNS - designed and performed experiments. ACCC - contributed reagents and expertise, DCS - participated in its design, participated in manuscript revision. WS - conceived of the study and contributed expertise. LAA - conceived of the study, analyzed data, and drafted the manuscript, and performed some dye injection experiments and Northern blotting experiments. All authors read and approved the final manuscript.
